# Patients’ engagement in primary care research: a case study in a Canadian context

**DOI:** 10.1186/s40900-020-00238-x

**Published:** 2020-11-01

**Authors:** Divya Kanwar Bhati, Michael Fitzgerald, Claire Kendall, Simone Dahrouge

**Affiliations:** 1 Bruyère Research Institute, Ottawa, ON Canada; 2 Department of Family Medicine, University of Ottawa, Ottawa, ON Canada; 3 Faculty of Medicine, University of Ottawa, Ottawa, ON Canada; 4 Li Ka Shing Knowledge Institute, St. Michael’s Hospital, Toronto, ON Canada; 5 Ottawa Hospital Research Institute, Ottawa, ON Canada

**Keywords:** Patient engagement, Primary care research, Evaluation, Canada

## Abstract

**Plain English summary:**

Patient engagement in primary care research is an increasingly common requirement, as it helps make research more relevant to patients and therefore more valuable. However, there is limited evidence about the outcomes on engagement and actually how it affects research. In Canada, the Canadian Institutes of Health Research has a Strategy for Patient-Oriented Research (SPOR), which in 2016 funded Ontario’s INSPIRE-PHC centre of excellence and its Patient Engagement Resource Centre (PERC). PERC conducted an online survey of the three INSPIRE-PHC studies that engaged patients to guide their research. We found that patient partners (PPs) were positive about their experience during research meetings, the value of collaboration, and the support that was provided. They were more involved in early stages of their research projects than in ongoing research activities. PPs valued their experience and also felt they had improved the research process and outcomes. This case study showed how PPs perceive their roles, but a more diverse group of PPs might have more differences in their experience.

**Abstract:**

**Background**

Patient engagement in primary care research is increasing and is now an expectation in many countries and funding agencies. In Canada, the Canadian Institutes of Health Research (CIHR) has mandated that patients be included as partners to guide the research process. Ontario’s Patient Engagement Resource Centre (PERC) was established in 2016 by the INNOVATIONS STRENGTHENING PRIMARY HEALTH CARE THROUGH RESEARCH (INSPIRE-PHC), one of 12 centres of excellence in the province funded under the CIHR’s Strategy for Patient-Oriented Research (SPOR) initiative. PERC’s mission is to support the authentic engagement of patients in primary care research. The present case study examines patients’ experience of engagement in INSPIRE-PHC research studies.

**Methods**

PERC conducted a web-based evaluation survey across the three INSPIRE-PHC studies that engaged patient partners (PPs). We used data collection tools developed by McMaster University (the Public and Patient Engagement Evaluation Tool (PPEET)) and the Patient-Centred Outcomes Research Institute (Ways of Engaging- ENgagement ACtivity Tool (WE-ENACT)) to assess patient experience and areas of involvement. These included both closed- and open-ended questions.

**Results**

The quantitative data showed that PPs were positive about their experience during research meetings, the value of collaboration, and the support that was provided to facilitate engagement. Most of them were highly involved in the initial stages of their research projects but much less involved in operational activities. The qualitative findings showed that, overall, PPs valued their experience, felt prepared to contribute and that their contributions were welcomed. In particular, they considered that they had improved the research process and outcomes. The majority also reported that they had learned from the experience and found it valuable.

**Conclusions**

This case study shows that patients engaged in three primary care research studies found the experience to be positive and felt that they had contributed to the research. This study adds to the literature on the evaluation of patient engagement in primary health care research. However, a study of a more diverse sample of PPs might elucidate differences in experience that could enrich future patient engagement activities.

## Background

Patient engagement in health research is now an expectation in many countries, and is actively fostered by funding agencies and charitable organizations [1–4]. A fundamental question where primary care research and health systems research is concerned is who researchers should seek to engage with. For disease-specific research such as arthritis [5], urinary tract infections [4], cancer or diabetes [6], it makes sense to engage patients with lived experience of the condition in question, and similarly for population-specific research, such as that involving the homeless [7] or adults with intellectual disabilities [8]. However, in primary health care and health systems research it is more difficult to know which patients to engage with, since these domains encompass the entire population. One step towards answering this question is to look at the actual experience of patient partners (PPs) engaged in primary care research projects. The present study explores the experience of five such PPs in Ontario, Canada.

As with research involving patient engagement, there are fewer studies of patient engagement that evaluate its impact in primary health care or health systems research. Some studies that look at these areas do so in the context of a broad range of health topics. For example, Hemphill et al. [9] surveyed 255 PPs across 139 research projects, including health systems projects, funded by the Patient-Centred Outcomes Research Institute (PCORI) in the U.S. to explore their motivations for engaging in research. Other studies that look at patients’ voice in the research process or the representativeness of patients engaged in research tend not to specify the research topic. For example, Maguire and Britten [10] interviewed 31 PPs across a range of health research studies in the UK to explore the concept of representation, but did not specify the topics of the studies. Similarly, Sieck, Hefner and McAlearney [11] surveyed 72 PPs and 81 academic researchers in the UK about their perspectives on patients as research partners, but again did not specify the topics of studies the PPs were involved in.

Patient engagement is well-established in the United Kingdom [12], but is more recent in North America. In the North American context, the term “patient” is usually used with an inclusive sense, comprising patients and family members/caregivers (e.g., Canadian Institutes of Health Research (CIHR) [13]), and sometimes patient advocacy organizations (e.g., Patient-Centered Outcomes Research Institute (PCORI) [14]). The Canadian Institutes of Health Research (CIHR) launched its Strategy for Patient Oriented Research (SPOR) in 2011, envisioning that “patients are active partners in health research that will lead to improved health outcomes and an enhanced health care system” [15]. Consequently, studies of patient engagement in research in Canada are quite recent. One such study evaluated engagement of patients and other stakeholders across 12 CIHR-funded Community-Based Primary Health Care (CBPHC) teams, and found that the experience of the project team in these engagement activities had been positive [16]. However, this study was unable to report separately on PPs’ perceptions of engagement. Another recent study in British Columbia with an explicit primary health care focus recruited 10 PPs from the BC Primary Health Care Research Network Patient Advisory to identify topics for future research, but did not report on the PPs’ *experience* of the priority setting activities [17]. In general, Canadian studies of patient engagement report predominantly on academic researchers’ experience [16, 18, 19].

The range of activities in which PPs are involved varies, depending on the stage of the research project. A meta-narrative systematic review of 142 papers conducted by PCORI on the spectrum of patient engagement showed that PPs were involved in choosing appropriate topics, improving enrollment rates, interpreting data, disseminating findings, and assisting researchers in securing funding [20]. Another meta-narrative systematic review described practical approaches to engaging patients in research, and concluded that patients could successfully play active roles as consultant (e.g., providing feedback on analysis or decisions), collaborator (e.g., participating in development of approaches to an issue), and partner (e.g., co-leading research and making decisions) [21, 22]. A narrative review of patient engagement literature in health and social care research also elaborated on values that patient partnerships add in terms of effectiveness, quality, reliability, representativeness and the evidence base for best practices in patient engagement in research [23]. Patients’ and caregivers’ motivations for engaging in research partnerships and the benefits they perceive have also been reported on. These include desiring to improve patient’s lives, providing support to healthcare interventions, feeling valued, making a positive difference, being empowered, and gaining new skills and better knowledge of research [6, 9, 24].

While the impact of patient engagement on study outcomes remains to be explored, a systematic review of studies conducted between 1995 and 2012 showed that researchers who engaged patient partners in their work reported gaining a better understanding of and building a better rapport with the community they were studying. However, some patient partners reported uncertainty about the concept of patient engagement in research [25] and feeling poorly prepared and trained to contribute, and others that their views were marginalized. Researchers faced difficulties in incorporating patient involvement due to their lack of ability to perceive PPs’ impact on research [26], or lack of money and time [1]. However, not all studies involving patients as co-researchers in health research result in better research, suggesting that collaborative efforts be given higher priority [27].

Ontario’s Patient Engagement Resource Centre (PERC) was established in 2016 by the INNOVATIONS STRENGTHENING PRIMARY HEALTH CARE THROUGH RESEARCH (INSPIRE-PHC) [28], one of 15 centres of excellence in the province funded under the CIHR’s SPOR initiative. This centre addresses the major health system challenges of equitable access to high quality primary health care (PHC) and better coordination of PHC with health and social care systems. PERC’s mission is to support the authentic engagement of patients in primary care research. PERC is a virtual entity that offers online ready access to resources, creates and delivers educational material, and provides mentorship related to patient engagement [29]. In 2016, INSPIRE-PHC was also funded by the Ontario Health Services Research Fund (HSRF) to undertake a program of research focused on primary care priorities, for which PERC was engaged to support any patient engagement activities related to the studies. Three of the seven INSPIRE-PHC studies engaged patient partners. These were intervention studies aimed at strengthening the linkage between primary health care, health systems and social sectors. PERC’s initial plan was to conduct both formative and summative evaluations annually to help the study teams evaluate the patients’ experience of engagement in research over a period of time. However, due to funding issues, study activities were brought to a halt, with the result that PERC conducted only one evaluation. The aim of this article is thus to report on the findings of the PERC evaluation. Out of 15 centres, INSPIRE-PHC focuses on PHC research, assisting researchers with primary health care-specific research consultation, and patient engagement resources. Hence, this study adds to the literature on the evaluation of patient engagement in PHC research supporting a larger network of interdisciplinary PHC researchers and decision makers.

## Methods

### Study design

The planned PERC support strategy consisted of conducting yearly formative evaluations of patient partners’ engagement activities in the INSPIRE-PHC projects’ research teams to identify areas for improvement, then offer support to the research team to address these gaps and to the patient partners to promote their ability to contribute to the research and enhance their own experience.

### Survey instrument

We adapted elements from the Public and Patient Engagement Evaluation Tool (PPEET) [30] and PCORI’s Ways of Engaging- ENgagement ACtivity Tool (WE-ENACT) [31] to assess patient experience and areas of involvement. The PPEET consists of three questionnaires (for participants, projects, and organizations) developed by a Canadian collaboration of researchers and public and patient engagement (PPE) practitioners led by McMaster University to evaluate public and patient engagement in health research [32]. The questionnaires were tested for suitability for implementation in a variety of health system settings, engagement activities, and user groups [33]. PCORI’s WE-ENACT was developed to describe the role of patients in research projects. The evaluation tool was assessed on its scientific rigour, taking into account the views of patient and public, comprehensiveness, and usability [34].

The adapted questionnaire developed by PERC assessed four dimensions of PPs’ experience using Likert scales: (i) experience during meetings (9 items); (ii) views on collaboration (9 items); (iii) appropriateness of support provided to engage (8 items); and (iv) roles and involvement in research (13 items). It also included five open-ended questions that explored PPs’ motivation to engage, key strengths of the collaboration, suggestions for improvement, training required, and contributions made to the research study (Appendix). The adapted survey questionnaire was reviewed by PERC’s patient advisory group members for clarity, ease of administration, time to completion and possible missing information.

### Recruitment

We invited the lead investigator and research coordinator of each study to identify their PPs and ask them if they were willing to complete the survey. Each team provided PERC with the contact information of the PPs who agreed to participate.

### Sampling and data collection

We emailed each PP to obtain their consent and to send a link to the survey on an encrypted web platform (SurveyMonkey). A follow-up email was sent after 1 week to remind partners to complete the survey. Out of seven patient partners involved in the three studies surveyed, five agreed to participate in the survey. We sent links to the survey by email to those patient partners who had agreed to participate to assess their engagement. Surveys were deployed, completed and analyzed between March, 2019 and May, 2019.

### Analysis

Raw and summary data were exported into MS Excel. Data with categorical responses were summarized using frequencies. Measures of scales were ranked using a Likert scale from 1 (strongly disagree) to 5 (strongly agree). The PERC team research lead and research fellow identified the key themes contained in the PCORI’s WE-ENACT questionnaire, and generated two broad categories of research cycle activities (oversight and operational) (Table 1). For open-ended responses, categories were extracted from the questions.

**Table 1:** Research Oversight and Operational activities

Oversight activities	Operational activities
Identifying research topics	Developing the budget
Developing the research question	Recruiting study participants
Proposal development	Data collection
Participating in the ethics process	Data analysis
Adding more people to the research team	Writing reports / summary
Study design	
Results review interpretation, or translation	
Sharing study findings	

## Results

Five of the seven (71.4%) INSPIRE-PHC patient partners responded and completed the survey. These PPs were between the ages of 60 and 80, and most were educated to university level. Most were also retired. Only two had experience of being a PP prior to joining their INSPIRE-PHC-funded project. They became involved in the research project through a connection with the researcher or another PP or community member, through being a volunteer, or through social media.

### Quantitative survey responses

#### At meetings with the research team

**Fig. 1:**
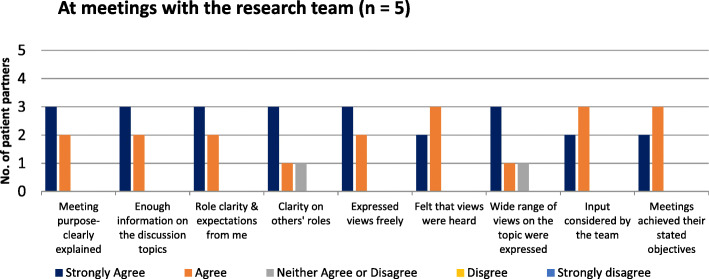
At meetings with the research team (*n* = 5)

Respondents were asked about their experiences during meetings with research team members. The patient experience was almost uniformly positive across all dimensions (Fig. 1).

#### Views on collaboration with the research team

**Fig. 2:**
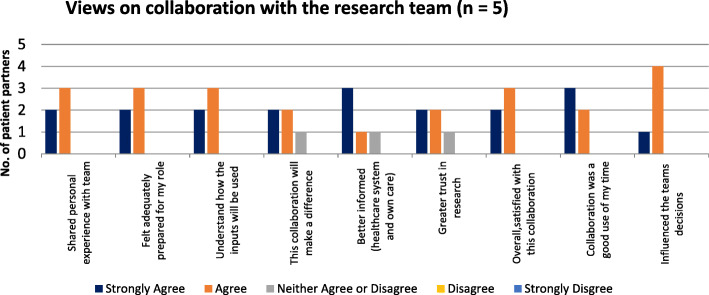
Views on collaboration with the research team (*n* = 5)

Respondents were asked to rate their agreement with statements about the research collaboration. Their perception of collaboration was again highly positive across all dimensions (Fig. 2).

#### Support provided by the research team

**Fig. 3:**
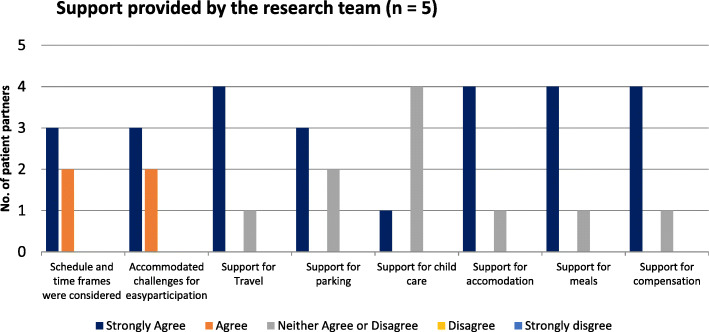
Support provided by the research team (*n* = 5)

Respondents were asked about the availability of the support they needed. Neutral responses were more prevalent, particularly in regards to support for child care, probably reflecting PPs needs (Fig. 3).

#### Patient partner’s roles and involvement with the research team

Respondents were asked which research activities they were involved in, in terms of oversight activities and operational activities.

##### Oversight activities (study initiation & review results)

**Fig. 4:**
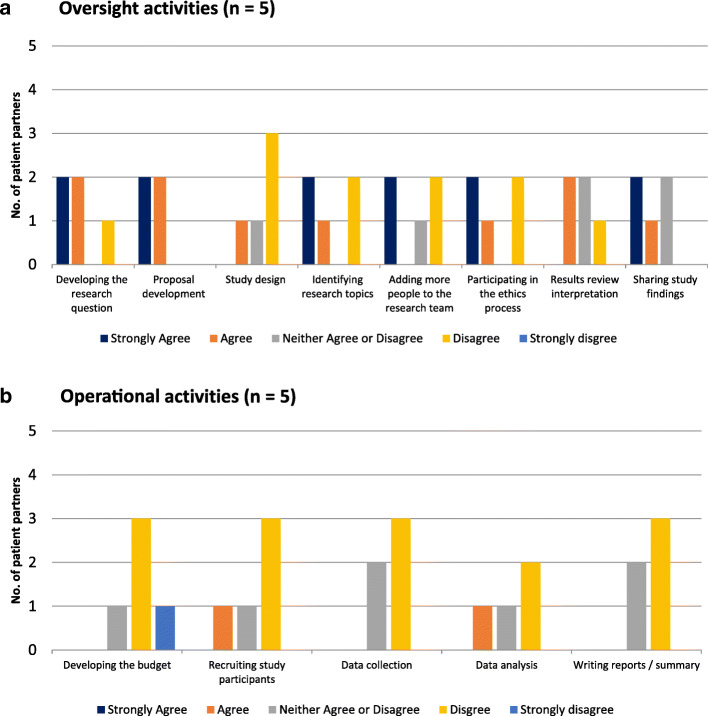
**a** Oversight activities (*n* = 5). **b** Operational activities (*n* = 5)

Respondents indicated divergent involvement in oversight activities, from uniform involvement in proposal development to mostly not being involved in study design. For other oversight activities, experiences varied (Fig. 4a).

##### Operational activities (study conduct activities)

Respondents indicated they had very little involvement in operational activities such as budgeting and data analysis (Fig. 4b).

### Qualitative findings

The key themes identified were: (1) motivation to engage; (2) strength of engagement; (3) influence on research; (4) improving engagement; and (5) training & support for engagement.

#### Motivation to engage

Among the reasons for their engagement in the research project, respondents indicated the influence of life experiences, and a desire to improve health conditions and provide care. One respondent expressed the sense of responsibility as a caregiver *“I felt the issue was really important, especially for my vulnerable loved one”* (PP3)*.* Engagement was also viewed as a way to influence the research and become a part of health care system “*I wanted to have an impact in the area of health care”* (PP5)*.*

#### Strengths of engagement

Respondents’ comments about the strengths of patient engagement focussed on (i) how it related to study outcomes, e.g., *“I have contributed my diverse experiences towards quality improvement in this study”* (PP4), and (ii) the scope it provided for interacting with other people with similar interests and experience. *“I enjoyed the variety of information shared from individuals with a wide range of insights”* (PP5)*.*

#### Influence on research

Most respondents felt that their participation had influenced the research project, whether its design (*“I think my contribution impacted the design of the intervention”* (PP4)), or its success (*“the study will achieve its goals through my engagement”* (PP2)). Overall, respondents thought they had contributed to a project that would improve access to and quality of care.

#### Improving engagement

Three respondents suggested areas where engagement could be improved: asking PPs about their needs ( “*the teams should ask what the patient hopes to get out of the experience, what issues brought them to the table, offer to open doors if needed to help patients connect with the people that may be able to help them address their concerns*” (PP5)); additional support for meetings (“*I need longer advance notice to attend the project meeting sessions*” (PP3)), and acknowledging PP contributions (“*I believe, recognition has to be given to moving from simply engaging partners for opinions after the fact rather that providing initial input to project design*” (PP4)).

#### Training and support required for engagement

Two respondents felt they were very well prepared to engage in research, one of whom commented positively on their experience attending the *“3-day Masterclass on patient engagement in research” (PP5).* Designed by the McMaster Health Forum and supported by the OSSU, Masterclass is an 11 week program for patients and families, health care providers, policymakers, and researchers to learn, conduct, and use patient oriented research [35]. However, two other respondents felt that the research team could have done more: *“I feel that, an initial lecture about the overall aims of the project long term and otherwise would have been helpful”* (PP2), and* “the research teams should ensure that partners fully understand the methodology and expected results and that more information on application of study results on policies and practices was required”* (PP5)*.* In contrast, another respondent said that *“I don’t see the need to learn more about research … my role was to provide the input necessary”* (PP1)*.*

## Discussion

This case study of patient partners’ experience of engagement in health research looked at a range of domains. In response to quantitative questions, PPs were positive about their experience during research meetings, the value of collaboration, and the support that was provided to facilitate engagement. Although most PPs were highly involved in the initial stages of their research projects, such as identifying research topics, developing the research question, proposal development and ethics processes, they were much less involved in operational activities such as budget development, study design, data collection, and data analysis. Responses to the qualitative questions about domains of experience showed that, overall, PPs valued their experience, felt prepared to contribute and that their contributions were welcomed. In particular, they considered that they had improved the research process and outcomes. Overall, the findings of our study are consistent with CIHR’s Strategy for Patient-Oriented Research [15], and thus add to the literature on evaluation of patient and stakeholder engagement in the Canadian context.

Our findings were also consistent with the PRioritiEs For Research (PREFeR) project that involved a group of patients as partners in a dialogue model [17]. Both PREFeR project and INSPIRE-PHC studies demonstrated a feasible approach to involving patients in setting research priorities in the context of primary health care research, although PREFeR focused on patient-friendly language that was used in the research and was agreed on by patient partners, whereas PPs in INSPIRE-PHC studies emphasized partners’ preparations to engage effectively, for example through an initial lecture about the project and complete understanding of the methodology and expected results. Other literature has also pointed out that the training needs and its uptake should be coherent with both researchers’ and patient partners’ perspectives [36].

This study adds to the small but growing literature on patients’ experience of engagement in research. Few studies that involve patient engagement actually incorporate evaluation of engagement from the outset, and even fewer involve patients in designing the evaluation to be used. The former is significant because it is possible that patients’ expectations and motivations change during the course of a research project (particularly if the project is a lengthy one) and, in retrospect, they may not always recall their initial perspective. The latter is important because, in the absence of patient engagement at *this* stage, patients are once again turned into the subjects of research rather than being researchers. We also conducted our evaluation towards the end of the INSPIRE-PHC studies and although PERC supported the patient engagement activities of these studies in several respects, we were not involved from the outset and therefore could not build the evaluation into the study designs.

In Canada, it is the CIHR’s mandate to include patients as partners to guide the research processes. Hence, primary care researchers are actively engaging and consulting patients in their research work. However, despite established patient engagement efforts, in this study, almost half of the research teams did not feel the need to include patients. This suggests that there might be an ongoing need to promote patient engagement and evaluation more broadly amongst primary care researchers in Canada. Hence, the study results also support the empirical literature on PPs perspectives as to what they contribute to actively enhancing research.

## Conclusions

Patient engagement evaluation is growing in Canada. However, primary healthcare studies that use standard evaluation tools to assess such engagement are limited. This case study uses standard patient engagement evaluation tools, and adds to the literature on the evaluation of patient engagement in primary health care research. Further studies that have a more diverse sample of PPs are required to enrich future patient engagement activities.

### Limitation and strengths

The small scale of this case study means that it cannot be taken to be representative of all PPs’ experience of engagement in research in Canada. In particular, our study participants are fairly homogeneous in terms of age, education, and work status. A study of a more diverse sample of PPs might elucidate differences in experience that could enrich future patient engagement activities. Despite these limitations, we believe that the results presented in this study provide insight into patient engagement in primary health care research in the Canadian context. Patient partner engagement has been successful in many research projects and has brought a unique added value and perspectives to the research. Although this is a relatively new approach in Canada that needs some adapting to, those researchers who have involved patient partners have come to recognize that it is meaningful if patient partners can contribute. On their part, when the research environment is conducive, patient partners are able to participate and to recognize the contribution they can make.

## Data Availability

The dataset generated and analysed during the current study is not publicly available due to confidentiality requirements, but is available in anonymized form from the corresponding author on reasonable request.

## Appendix

### Data collection tool

We often refer to such team members as “Patient Partners”, although we recognize that some may not be patients themselves, but perhaps a caregiver for an individual. By “Patient Partners”, we also mean who have lived experience and who have been engaged in the research work. Bruyère Research Institute’s Investigator Dr. Simone Dahrouge is part of a group of seven research teams known as INSPIRE-PHC-2 i.e. Innovations Supporting Primary Healthcare through Research who are working on projects to ameliorate the health services patients receive. To foster meaningful patient engagement across all the seven research studies and as part of our commitment to the Canadian Institutes of Health Research (CIHR) to support patient engagement in research, the PERC initiative was launched to support the resource needs of the researchers and patient partners. Hence, this work is a part of a broader initiative that aims to promote authentic patient engagement in primary health care research. That initiative is the Patient Engagement Resource Centre (PERC): (https://www.patientengagement-phcresearch.com).

#### About the evaluation survey

The PERC team is in the process of conducting an online patient engagement evaluation survey, the components are adapted from a validated patient engagement evaluation tool that aims to understand the experience of Patient Partners who are working on one of the seven health services research projects. It will also help the PERC team to understand how the teams can support Patient Partners in their efforts to inform the research work in which they are involved. We encourage you to discuss the questions with your team members, to ensure that the answers are as representative as possible. Completion of this survey implies that you have consented to participate in our research study.

We estimate the survey should take approximately 45 min to complete.

#### Instructions to complete the survey

- We are interested in your feedback about the research project engagement activity that you recently participated in.
- The questionnaire is composed of several statements. Please indicate your level of agreement with each statement and check only one box for each statement.
- Please provide additional feedback in the open- ended question boxes provided in the end of this questionnaire.

Your responses to these questions will be kept strictly confidential. Your responses will be identified by a participant number which is not linked to the project in which you are involved. Only summary data will be shared with the research teams from these seven projects. In this regard, please share your responses. Please note completing the survey is ‘voluntary’.

If you have any questions, please do not hesitate to contact the PERC lead Dr. Simone Dahrouge at 613–562-6262, Ext. 2913 or sdahrouge@bruyere.org or Divya Bhati at dbhati@bruyere.org.

1. **Experience with the Collaboration**

This section is composed of several statements. Please indicate your level of agreement with each statement by rating the statement according to the following scale:

**Table:**

Statements	Strongly Agree	Agree	Neither Agree nor Disagree	Disagree	Strongly Disagree
**a. At our meetings with the researchers:**					
The purpose of the meetings was clearly explained.					
I had enough information to contribute to the topics being discussed.					
I was clear about my role and what was expected of me					
I had clarity on the role of other team members					
I was able to express my views freely.					
I felt that my views were heard.					
A wide range of views on the topic were expressed.					
I felt that the input provided will be considered by the team.					
The meetings achieved their stated objectives.					
**b. Overall views when thinking about my collaboration with this team:**					
My insights and comments influenced the decisions of the team.					
I was able to contribute and share my personal experience with this team.					
I felt adequately prepared for my role.					
I understand how the input from this collaboration will be used.					
I think this collaboration will make a difference.					
As a result of my collaboration with this team, I am better informed about the healthcare system and issues that can affect my own care					
As a result of my collaboration with this team, I have greater trust in research and the efforts made to improve our healthcare system					
Overall, I was satisfied with my collaboration with this team.					
My collaboration with this team was a good use of my time.					
**c. Logistics, Expenses & Compensation:**					
My schedule and timeframes were considered when the team meetings were scheduled.					
The research team worked with me to accommodate my challenges and ensure that it is easy for me to participate.					
I feel the support I needed for my travels were available.					
I feel the support I needed for my child care was available.					
I feel the support I needed for my travels were available.					
I feel the support I needed for my accommodations were available.					
I feel the support I needed for my meals were available.					
I feel the support I needed towards my participation remuneration / compensation was available.					

2. **Understanding roles and involvement:**

This section is composed of several statements. I was involved in the following activities of the research cycle. Please indicate your level of agreement with each statement by rating the statement according to the following scale:

**Table:**

Statements	Strongly Agree	Agree	Neither Agree nor Disagree	Disagree	Strongly Disagree
Identifying research topics					
Developing the research question					
Proposal development					
Developing the budget					
Participating in the ethics process					
Adding more people to the research team					
Study design					
Recruiting study participants					
Data collection					
Data analysis					
Results review interpretation, or translation					
Sharing study findings					
Writing reports / summary					
Other activity: please specify					

#### Open-ended questions

Please feel free to express your views on different aspect of your participation such as; motivation to participate, improvement needed, any training required and overall use of your contribution.

1. What motivated you to be a part of this research project initiative?
2. What was the best thing about this engagement activity?
3. Please identify at least one improvement that the project team could make for their future engagement activities.
4. What preparation (any orientation / training / presentation) would you have liked to have had for this partnership with research team? *(Example: information about research process in general, your role on the team, any ethical conduct training, and information about the specific area of research)*
5. How do you think your contribution will be used in the study?

### Demographic information (optional)

In this section, please help us understand a bit about yourself. The following is a list of ‘**optional**’ socio-demographic questions that may be useful to include as part of the participant evaluation where there is minimal prior knowledge about participants and where there is an interest in gaining a deeper understanding about participant backgrounds and the extent to which they represent communities affected by and relevant to the topic or issue that is the focus of the engagement activity.

1. In which year were you born? (enter 4-digit birth year; for example, 1976)
2. What is the highest level of education that you have completed?
  - No schooling
  - Completed elementary school
  - Completed high school
  - Completed community college
  - Completed technical college
  - Completed Bachelor’s degree (Arts, Science)
  - Completed post graduate course / training
3. What is your work status?
  - Unable to work
  - Home maker
  - Student (working part-time)
  - Retired
  - Working part-time
  - Working full time
4. Have you had prior experience working as a Patient Partner with another research team(s)?
  - Yes
  - No
5. Please tell us more about yourself: *(Background, work, interests)*
6. How did you come to work with the research team?
  - Through a patient organization to which I belong
  - Through a personal connection I have with a research team member
  - Other (specify)

Thank you for taking the time to complete this survey. Your responses have been recorded. On behalf of the PERC team, we thank you for your contribution to helping us understand the nature, extent and experience of patient engagement in research.

## Abbreviations

CIHR: Canadian Institutes of Health Research
PERC: Patient Engagement Resource Centre
PCORI: Patient Centered Outcomes Research Institute
SPOR: Strategy for Patient Oriented Research
